# Supplementary material for: The Diagnostic Value of Circulating Cell-Free HPV DNA in Plasma from Cervical Cancer Patients
Source: Cells. 2022 Jul 11;11(14):2170. doi: 10.3390/cells11142170 (PMC9315636; doi:10.3390/cells11142170)

Supplementary material for "The diagnostic value of circulating cell-free HPV DNA in plasma from cervical cancer patients"

Sara Bønløkke [1,2,*], Magnus Stougaard [1,2], Boe Sandahl Sorensen [1,3], Berit Bargum Booth [4], Estrid Høgdall [5], Gitte-Bettina Nyvang [6], Jacob Christian Lindegaard [7], Jan Blaakær [8,9], Jesper Bertelsen [2], Katrine Fuglsang [4], Mikael Lenz Strube [10], Suzan Lenz [11] and Torben Steiniche [1,2]

Table S1. Clinical and biological data on the overall study cohort

Early-stage subgroup

Pt no	Age	Histology[1]	FIGO 2018[2]	T-score[3]	Tumor size (mm)[4]	Treatment[5]	Re-staged[6]	HPV[7]	ccfHPV DNA (copies/ml)[8]	ccHPV DNA pos/neg as by cutoff[9]
1	46	ASC	IB2	2	20	Rad hys+PL+BSO		HPV 18	0	Neg
2	36	AC	IB1	1	10	Rad hys+PL+BSO		HPV 18	0	Neg
3	44	ASC	IB1	2	26	Rad hys+PL+BSO	IB2	HPV 18	0	Neg
4	36	AC	IB1	2	24	Rad hys+SLN+BSO	IB2	HPV 16	1	Neg
5	41	SCC	IB1	1	8	Rad hys+SLN+BSO		HPV 16	0	Neg
6	47	SCC	IA2	0.5	7,5	Rad hys+SLN+BSO		HPV 16	0	Neg
7	43	SCC	IA2	0.5	10	Rad hys+SLN+BSO		HPV 16	0	Neg
8	57	SCC	IB2	2	22	Rad hys+SLN+BSO		HPV 16	0	Neg
9	73	SCC	IB2	2	10	Rad hys+SLN+BSO		HPV 16	0	Neg
10	51	SCC	IA2	0.5	9	Sim hys+PL+BSO		HPV 16	1	Neg
11	36	SCC	IB1	2	25	Rad hys+SLN+BSO	IB2	HPV 16	0	Neg
12	72	AC	IA2	0.5	7,2	TLH+PL+BSO		HPV 16	0	Neg
13	40	AC	IB1	1	19	Rad hys+SLN+BSO		HPV 16	2	Neg
14	30	SCC	IA2	0.5	14	Rad hys+SLN+BSO		HPV 18	0	Neg
15	41	AC	IB1	1	8,1	Rad hys+SLN+BSO		HPV 18	0	Neg
16	47	SCC	IA2	0.5	8	Rad hys+SLN+BSO		HPV 18	0	Neg
17	43	SCC	IB2	1	12	Rad hys+SLN+BSO	IB1	HPV 16	3	Neg
18	33	SCC	IA1	0.25	17	Collum amputation+SLN		HPV 16	0	Neg

19	44	AC	IA2	0.5	12	Rad hys+SLN+BSO		HPV 18	0	Neg
20	83	SCC	IB1	1	10	Rad hys+SLN+BSO		HPV 16	0	Neg
21	39	AC	IB1	1	18	Rad hys+SLN+PL+BSO+ Omenectomy		HPV 18	0	Neg
22	44	AC	IB2	2	22	Rad hys+SLN+BSO		HPV 16	1	Neg
23	34	SCC	IB2	2	20	Rad hys+SLN+BSO		HPV 16	1	Neg
24	50	AC	IA2	0.5	8	Rad hys+SLN+BSO		HPV 16	1	Neg
25	66	AC	IA2	0.5	10	Rad hys+SLN+BSO		HPV 16	1	Neg
26	33	AC	IA2	0.5	13	Rad hys+SLN+salpingo-oophorectomy dxt		HPV 16	13	Pos
27	63	AC	IB1	2	28	Rad hys+SLN+BSO	IB2	HPV 18	0	Neg
28	40	AC	IB2	2	24	Rad hys+SLN+BSO		HPV 16	2	Neg
29	33	SCC	IB1	1	18	Rad hys+SLN+BSO		HPV 16	5	Pos
30	56	SCC	IB1	2	23	Rad hys+SLN+BSO	IB2	HPV 16	4	Pos
Late-stage subgroup										
Pt no	Age	Histology	FIGO 2018	T-score	Tumor size (mm)	Treatment	Re-staged	HPV	ccfHPV DNA (copies/ml)	ccHPV DNA pos/neg as by cutoff
31	60	AC	llB	3	35	EBRT+BT+Cis	N/A[10]	HPV 16	24	Pos
32	72	SCC	llB	3	40	EBRT+BT+Cis	N/A	HPV 16	541	Pos
33	56	SCC	llB	4	50	EBRT+BT+Cis	N/A	HPV 16	88	Pos
34	49	SCC	IIIC1	5	40	EBRT+BT+Cis	N/A	HPV 16	41	Pos
35	53	SCC	IIB	4	40	EBRT+BT+Cis	N/A	HPV 16	0	Neg
36	50	SCC	IIB	6	50	EBRT+BT+Cis	N/A	HPV 16	2	Neg
37	61	SCC	IIIC1	9	60	EBRT+BT+Cis	N/A	HPV 16	186	Pos
38	85	SCC	IIB	4	50	EBRT+BT	N/A	HPV 16	54	Pos
39	40	SCC	IIB	3	5	EBRT+BT+Cis	N/A	HPV 16	0	Neg
40	42	SCC	IIIC1	3	35	EBRT+BT+Cis	N/A	HPV 18	1.5	Neg
41	51	SCC	IIB	7	65	EBRT+BT+Cis	N/A	HPV 16	76	Pos
42	41	SCC	IIIC1	7	55	EBRT+BT+Cis	N/A	HPV 16	0	Neg
43	35	SCC	IIIC1	2	24	EBRT+BT+Cis	N/A	HPV 16	1	Neg
44	46	SCC	IIIC1	3	35	EBRT+BT+Cis	N/A	HPV 18	0	Neg
45	55	AC	llB	6	52	EBRT+BT+Cis	N/A	HPV 16	4	Pos

46	59	SCC	IVA	17	120	Neoadjuvant Taxol/Topotecan/ Avastin followed by EBRT+BT	N/A	HPV 16	0	Neg
47	30	SCC	IIIC1	5	60	EBRT+BT+Cis	N/A	HPV 16	28	Pos
48	77	SCC	IIIC1	5	45	EBRT+BT+Cis	N/A	HPV 16	58	Pos
49	46	SCC	IIIC1	5	50	EBRT+BT+Cis	N/A	HPV 16	1	Neg
50	52	AC	IIB	3	40	EBRT+BT+Cis	N/A	HPV 16	0	Neg
51	43	SCC	IIB	3	42	EBRT+BT+Cis	N/A	HPV 16	43	Pos
52	57	SCC	IIIC1	5	40	EBRT+BT+Cis	N/A	HPV 16	30	Pos
53	27	SCC	IIIC2	10	70	EBRT+BT+Cis	N/A	HPV 16	4	Pos
54	55	SCC	IIB	7	45	EBRT+BT+Cis	N/A	HPV 16	181	Pos
55	53	SCC	IB3	3	40	EBRT+BT+Cis	N/A	HPV 16	9	Pos
56	40	SCC	IIIC1	9	100	EBRT+BT+Cis	N/A	HPV 16	11	Pos
57	41	SCC	IIIC1	3	70	EBRT+BT+Cis	N/A	HPV 16	208	Pos
58	32	SCC	IB3	3	45	EBRT+BT+Cis	N/A	HPV 16	136	Pos
59	57	SCC	IIIC1	11	80	EBRT+BT+Cis	N/A	HPV 18	0	Neg
60	55	AC	IIIC2	10	100	Neoadjuvant Cisplatin followed by EBRT+BT+Cis	N/A	HPV 16	810	Pos

[1] Abbreviations: Squamous cell carcinoma (SCC), adenocarcinoma (AC), adenosquamous carcinoma (ASC)

[2] Disease stage according to FIGO 2018 (Bhatla, N., et al., *Revised FIGO staging for carcinoma of the cervix uteri.* Int J Gynaecol Obstet, 2019. 145(1): p. 129-135.). For early-stage patients having been re-staged after surgery, the re-staged stage is the one listed.

[3] T-score according to Lindegaard, J.C., et al., *Prognostic implications of uterine cervical cancer regression during chemoradiation evaluated by the T-score in the multicenter XXX study.* Int J Radiat Oncol Biol Phys, 2022. The scoring system is developed for cervical cancer patients with stage IB-IVB disease, giving patients with stage IB1, IB2, and IB3 a T-score of 1, 2, and 3 respectively. Thus, for patients with stage IA1 and IA2 disease, we made a presumption that stage IA1 equals a T-score of 0.25 and stage IA2 equals a T-score of 0.5. For early-stage patients having been re-staged after surgery, the re-staged stage is the one used to determine T-score.

[4] Larges diameter of tumor. For the early-stage subgroup, tumor size was evaluated pathologically *after* surgery based on the removed tissue. For the late-stage subgroup, tumor size was evaluated based on clinical examination or magnetic resonance imaging (MRI) prior to treatment.

[5] Treatment abbreviations:

- Rad hys+PL+BSO = Radical hysterectomy (Rad hys) with pelvic lymphadenectomy (PL) and bilateral salpingo-oophorectomy (BSO).
- Rad hys+SLN+BSO = Radical hysterectomy with sentinel lymph node (SLN) removal and bilateral salpingo-oophorectomy.
- Sim hys+PL+BSO = Simple hysterectomy (sim hys) with pelvic lymphadenectomy and bilateral salpingo-oophorectomy.
- TLH+PL+BSO = Total laparoscopic hysterectomy (TLH) with pelvic lymphadenectomy and bilateral salpingo-oophorectomy.
- EBRT+BT+Cis = External beam radiation therapy with brachytherapy and concomitant weekly cisplatin intravenously.

[6] Pathological re-staging *after* surgery.

[7] Cervical tissue biopsy tested with INNOLiPA® Genotyping Extra II (Fujirebio)

[8] Blood sample collected prior to treatment analyzed for HPV DNA with ddPCR. Samples were analyzed in triplicates, and the results represent the mean value.

[9] Cutoff for ccfHPV DNA positivity is > 3 copies/ml plasma.

[10] Not applicable.

**Table S2. Causes for exclusion from study**	
**Cause**	**Number of patients**
For patients excluded prior to treatment	*81*
Did not wish to participate	10
Recurrence of a prior cervical cancer	25
Referred directly to palliative treatment	3
Metastasis from other primary cancer	1
Pathological revision of cervical tissue biopsy showed FIGO<IB1 (from June 2018 to December 2019 with FIGO 2009[1] and from January 2020 to October 2020 with FIGO 2018[2])	17
No malignancy detected by pathological review	1
Dementia or severe comorbidity	6
Did not speak Danish	5
Psychiatric problems	4
Missed (not asked)	9
For patients excluded after final staging	*39*
Withdrew consent before treatment initiation	5
Referred to palliative treatment due to disease stage	7
Pathological revision at multidisciplinary team (MDT) conference showed endometrial cancer	2
Pathological revision at MDT conference showed vaginal or vulvar cancer	3
Final staging after MDT conference, clinical examination, PET CT and MRI scan showed FIGO stage <IB1 (from June 2018 to December 2019 with FIGO 2009[1] and from January 2020 to October 2020 with FIGO 2018[2])	15
Not enough tissue material for analysis	1
Refused to receive treatment	1
HPV negative cervical tissue	5

[1] Quinn, M., et al., *Carcinoma of the cervix uteri.* International Journal of Gynecology & Obstetrics, 2006. **95**: p. S43-S10

[2] International Federation of Obstetrics and Gynecology, FIGO 2018 (Ref: Bhatla, N., et al., *Revised FIGO staging for carcinoma of the cervix uteri.* Int J Gynaecol Obstet, 2019. 145(1): p. 129-135.)

Table S3. Primer pairs and corresponding probes for the ddPCR analyses			
Primer name	Sequence (5'→3')	Sequence probe (5'→3')	Amplicon size (bp)
HPV16 E7 Forward primer	TCC AGC TGG ACA AGC AGA AC	FAM - ACA GAG CCC ATT ACA AT	83
HPV16 E7 Revers primer	CAC AAC CGA AGC GTA GAG TC		
HPV18 E7 Forward primer	CAA CAT TTA CCA GCC CGA C	VIG - AAC CAC AAC GTC ACA CAA	71
HPV18 E7 Revers primer	CTG GCT TCA CAC TTA CAA CAC		

Table S4. Concentration (copies/ml) of ccfHPV16/18 DNA detected by ddPCR in four cervical cancer tissue samples[1], eight plasma samples from CIN3 patients, and 15 plasma samples from healthy controls.	
Cervical cancer tissue samples (n = 4)[2]	ccfHPV DNA (copies/ml)[3]
1 (HPV16)	9675
2 (HPV16)	19356
3 (HPV18)	12629
4 (HPV18)	2621
CIN3 plasma samples (n = 8)[2]	
1 (HPV16)	0
2 (HPV16)	0
3 (HPV16)	1.33
4 (HPV16)	1.33
5 (HPV16)	0.33
6 (HPV16)	0
7 (HPV16)	0
8 (HPV16)	0.67
Healthy control plasma samples (n = 15)[2]	
1 (HPV16/18)	1/1.33
2 (HPV16/18)	0/3
3 (HPV16/18)	0.67/1.67
4 (HPV16/18)	0.33/0.33
5 (HPV16/18)	0.33/0.33
6 (HPV16/18)	0.66/0
7 (HPV16/18)	0/0.33
8 (HPV16/18)	1.33/0
9 (HPV16/18)	0.33/0
10 (HPV16/18)	0.33/0.67
11 (HPV16/18)	0.67/1.67
12 (HPV16/18)	1.67/3
13 (HPV16/18)	0.67/1.33
14 (HPV16/18)	0.33/1.33
15 (HPV16/18)	1/1

[1] The cervical cancer samples analyzed stem from cervical tissue from four of the included patients; two HPV 16 positive patients and two HPV 18 positive patients (tested with INNO-LiPA HPV Genotyping Extra II assay (LiPA) (Fujirebio Europe, Ghent, Belgium).

[2] The HPV genotype in brackets is the type tested for with ddPCR. For cervical cancer samples (positive controls) and the CIN3 plasma samples (CIN3 controls), the type tested for is the same as detected in the cervical tissue sample from the same participant (tested with INNO-LiPA HPV Genotyping Extra II assay (LiPA) (Fujirebio Europe, Ghent, Belgium). Healthy control samples were tested for both HPV genotypes (HPV16/HPV18), and results on ccfHPV DNA therefore constitute values for ccfHPV 16 DNA and ccfHPV18 DNA, respectively.

[3] Samples were analyzed in triplicates, and the results represent the mean value.

Figure S1: Inclusion of HPV associated CIN3 patients.

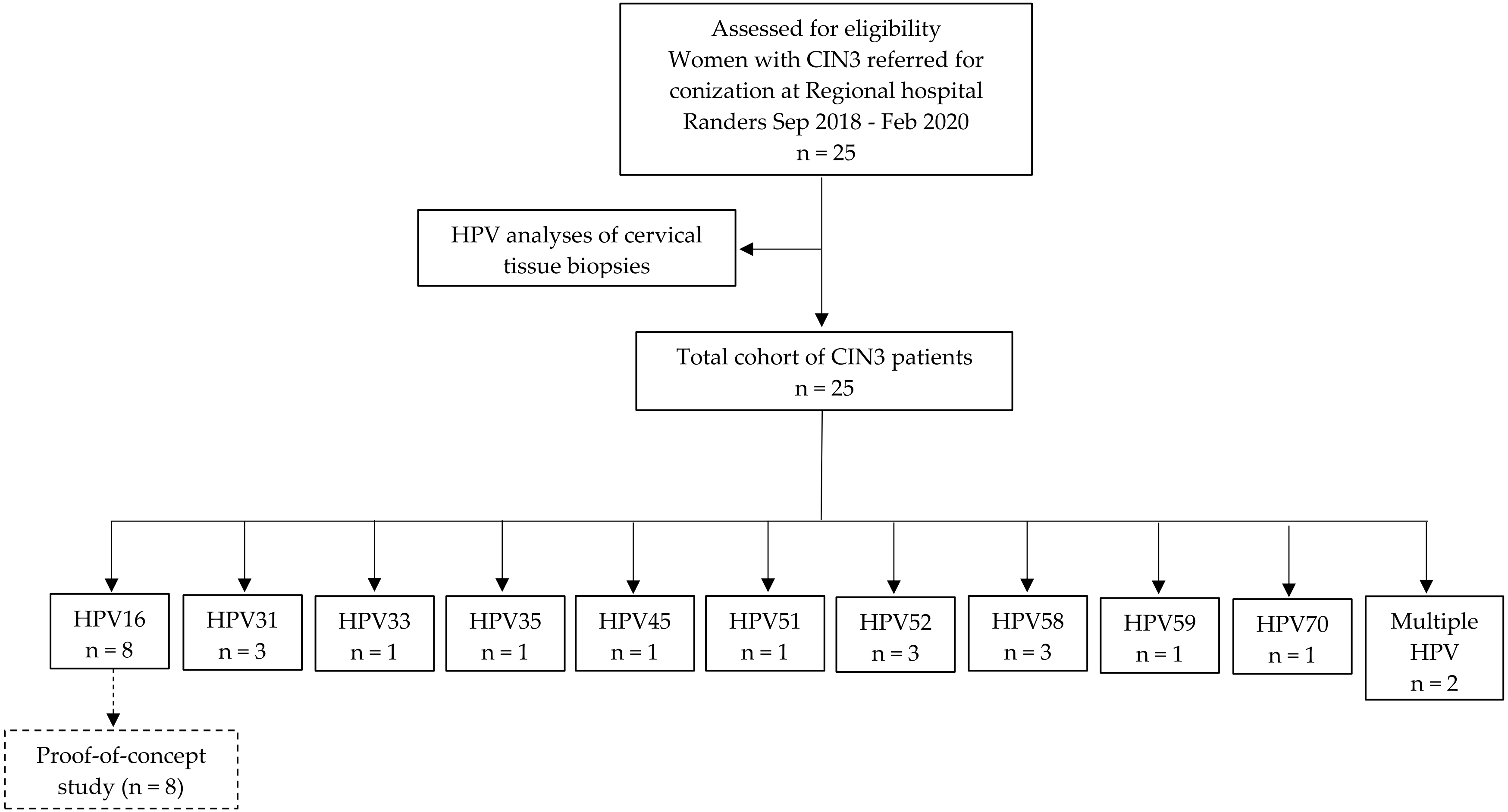

Supplement: Supplementary file 1 [file cells-11-02170-s001.zip › cells-1805937-supplementary.pdf]
